# Involvement of Normalized Glial Fibrillary Acidic Protein Expression in the Hippocampi in Antidepressant-Like Effects of Xiaoyaosan on Chronically Stressed Mice

**DOI:** 10.1155/2017/1960584

**Published:** 2017-03-02

**Authors:** Xiu-Fang Ding, Yan Liu, Zhi-Yi Yan, Xiao-Juan Li, Qing-Yu Ma, Zhong-Ye Jin, Yan Liu, Yue-Hua Li, Yue-Yun Liu, Zhe Xue, Jia-Xu Chen, Zhi-Ping Lv

**Affiliations:** ^1^School of Basic Medical Science, Beijing University of Chinese Medicine, Beijing 100029, China; ^2^Henan University of Chinese Medicine, Henan, China; ^3^School of Pre-Clinical Medicine, Hubei University of Chinese Medicine, Wuhan, China; ^4^Beijing Chaoyang Hospital, Capital Medical University, Beijing, China; ^5^School of Traditional Chinese Medicine, Southern Medical University, Guangzhou, Guangdong, China

## Abstract

The research has only yielded a partial comprehension of MDD and the mechanisms underlying the antidepressant-like effects of XYS. Therefore, in this study, we aimed to explore the effects of XYS on chronic unpredictable mild stress- (CUMS-) induced changes in the neuronal and the astrocytic markers in the mouse hippocampus. The physical states and depressive-like behaviors in mice with CUMS were recorded. The serum contents of brain-derived neurotrophic factor (BDNF) and glial cell line-derived neurotrophic factor (GDNF) were measured. The protein and mRNA expressions and the immunoreactivities of glial fibrillary acidic protein (GFAP) and neuronal nuclei (NeuN) in mouse hippocampus were detected using a Western blot, qRT-PCR, and immunohistochemical staining, respectively. XYS treatment markedly improved the physical state and depressive-like behaviors in mice subjected to CUMS compared with the model group, and the serum contents of BDNF and GDNF were significantly upregulated. XYS treatment also elevated the protein and mRNA levels, as well as the immunoreactivity of GFAP in the hippocampus. However, CUMS did not influence NeuN expression. In conclusion, these results reveal that chronic administration of XYS elicits antidepressant-like effects in a mouse model of depression and may normalize glial fibrillary acidic protein expression in the hippocampi of mice with CUMS.

## 1. Introduction

Major depressive disorder (MDD), also called major depression (MD), is a highly prevalent chronic illness and a common psychiatric disorder affecting a rising percentage of the world's population. According to the World Health Organization, depression may become one of the main leading causes of disease burden by 2030 [1]. This mental disorder presents with depressed mood, disturbed appetite or sleep, feelings of guilt or low self-worth, loss of interest, fatigue, and difficulty making decisions or poor concentration.

Till date, it has been recognized that neuronal deficits are insufficient to explain the pathophysiology of neuropsychiatric disorders and the action mechanism of antidepressant drugs. Increasing evidence suggests an important role for glial changes in the fundamental processes related to the development and treatment of MDD. In preclinical research, animal models of chronic stress are a valuable tool for studying the molecular mechanisms that may lead to MDD [2]. The effects of chronic social stress on neurons include reduced neurogenesis in the subgranular zone of the dentate gyrus [3] and reduced expression of glycoprotein M6 in the axonal membrane of glutamatergic neurons [4, 5]. Chronic social stress also affects glial cells in the hippocampus of male tree shrews, where it decreases the number of immunocytochemically detectable astrocytes [6]. Thus, the changes in neurons or astrocytes in the hippocampus are involved in the pathogenesis of MDD.

It was recently reported that only one-third of patients with MDD experience a complete therapeutic improvement using currently available antidepressant drugs, with a therapeutic effect appearing only after several weeks of treatment. Thus, the discovery of effective antidepressants is urgently needed. Many Chinese herbal medicines have antidepressant-like effects [7–11]. Among them, Xiaoyaosan (XYS) is the most commonly used prescription for the treatment of depression and has been widely used for thousands of years since the Song dynasty. According to traditional Chinese medical (TCM) theories, patients with MDD usually develop liver stagnation and spleen deficiency. XYS can soothe the liver, invigorate the spleen, and nourish the blood; therefore, it can be used to treat MDD. A systematic review found that XYS was more effective than placebo and had effects comparable to antidepressants; however, it should be noted that the studies were of poor methodological quality due to study design and variation in the different types of herbal formulas [9]. XYS is the most frequently prescribed herbal formula for treating major depression [12]. A clinical research study showed that XYS can significantly decrease the Hamilton depression scores in patients with MDD. In addition, XYS treatment can effectively improve depressive-like behaviors in rats exposed to chronic immobilization stress (CIS) through the inhibition of activity in the locus ceruleus/norepinephrine (LC/NE) systems [8].

The important characteristics of Traditional Chinese Medicine (TCM) prescriptions are that they consist of multiple constituents, utilize multiple pathways, and have multiple targets. However, little is known about whether XYS has therapeutic effects on the hippocampus that directly involve either neuronal or astrocytic markers. Therefore, based on the previous studies, the present study aims to investigate the effects of XYS on CUMS-induced changes in the neuronal marker NeuN and the astrocytic marker GFAP in the hippocampus. Furthermore, the effects of XYS on CUMS-induced changes in physical state, depressive-like behaviors, and neurotrophic factors (BDNF and GDNF) were also measured.

## 2. Materials and Methods

### 2.1. Preparation of Drugs

The XYS prescription consists of eight herbal medicines: Poria [*Poria cocos* (Schw.) Wolf], Rhizoma Zingiberis Recens (*Zingiber officinale* Rosc.), Radix Angelicae Sinensis [*Angelica sinensis* (Oliv.) Diels], Radix Bupleuri (*Bupleurum chinense* DC.), Rhizoma Atractylodis Macrocephalae (*Atractylodes macrocephala* Koidz.), Radix Paeoniae Alba (*Paeonia lactiflora *Pall.), Radix Glycyrrhizae (*Glycyrrhiza uralensis* Fisch.), and Herba Menthae (*Mentha haplocalyx* Briq.). These raw herbs were purchased from the Tongrentang (Bozhou, AnHui) Decoction Pieces Limited Company and then authenticated by Dr. B. Liu of the Beijing University of Chinese Medicine. The drugs were extracted by the Chinese medicine preparation room of the China-Japan Friendship Hospital as described previously [13]. The extraction rate was 18.8%, and the quality of XYS was identified by high-performance liquid chromatography-mass spectrometry analysis (LC-MS/MS) [14].

### 2.2. Animals and Experimental Procedures

The healthy C57BL/6J male mice (age: 12 weeks) were purchased from Beijing Vital River of the Charles River company (number SCXK 2011-0004). All animals were housed within a standard animal room (22 ± 1°C, 12 h/12 h dark/light cycle, relative humidity: 30%–40%). The protocol in this experiment was approved by the Animal Ethics Committee of Beijing University of Chinese Medicine and was in accordance with all guidelines for the Care and Use of Laboratory Animals of China. The mice were allowed to acclimate for one week before the onset of experiments.

A total of 60 mice were randomly divided into four groups according to their body weights. The four groups were control group (no stress + physiological saline), model group (CUMS + physiological saline), Xiaoyaosan treatment group (CUMS + XYS), and FLU treatment group (CUMS + FLU). All mice were individually housed in cages. The mice in the model group and the two treatment groups underwent CUMS for 21 days. The CUMS paradigm is shown in Table 1. Mice in the control group and model group received 0.5 mL distilled water by intragastric administration. Mice in the two treatment groups were given 0.25 g/kg/d XYS and 2.6 mg/kg/d FLU, respectively. The dose of XYS (0.25 g/kg/d) was selected on the basis of its satisfactory efficacy previously described elsewhere [15, 16].

### 2.3. Coat State and Body Weight in Mice Exposed to CUMS

To investigate the dynamics of the CUMS response, the coat state and the body weight of each mouse were evaluated. The coat state and body weight were recorded weekly until the end of CUMS (day 21). As previous reported [17], the total score of the coat state in this experiment was calculated as the sum of scores from 7 different body parts, including the head, neck, dorsal coat, ventral coat, forepaws, tail, and hind paws. For each body area of the mouse, a score of 1 was recorded for a well-groomed coat and a score of 0 was recorded for an unkempt coat.

### 2.4. Behavior Tests

The open field test (OFT) was used to estimate locomotor activity and anxiety-like behavior in a dimly lit and quiet room. Testing was performed in a four-sided (40 × 40 × 15 cm) wooden enclosure with the walls painted black and the floor was divided into 25 equal squares by blue lines. The mouse was placed gently in the center and left to explore the area for 5 min. For scoring, a video-tracking system (EthoVision, Noldus, Wageningen, Holland) was used to record the total running distance (locomotor activity) and number of entries into the center zone.

The 5-day sucrose preference test (SPT) protocol was performed as previously reported [18, 19]. In brief, mice were housed individually and habituated to two bottles filled with tap water on days 1-2 and 1% sucrose solution (Biotech, #0335) on days 3-4. On day 5, mice were given a free choice between two bottles for 48 h, one with tap water and the other with 1% sucrose solution. The position of the two bottles was changed after 12 h to eliminate side preference in drinking behavior. No water or food deprivation was applied before the test. The sucrose preference was calculated as the percentage of the 1% sucrose solution consumed out of the total amount of liquid consumed.

### 2.5. Brain-Derived Neurotrophic Factor (BDNF) and Glial Cell Line-Derived Neurotrophic Factor (GDNF) Measurement

The experiment was terminated on the 21st day, and mice were anesthetized with an intraperitoneal injection of 3% sodium pentobarbital (30 mg/kg). Blood samples were collected from the ocular artery and placed into 1.5 mL EP tubes. After leaving them at room temperature for 30 min, the samples were centrifuged for 10 min at 3,000 ×g. Serum was then used to measure BDNF and GDNF levels using a microtiter plate reader (Victor3V, Perkin Elmer, Waltham, MA, USA) according to the protocol in the enzyme-linked immunosorbent assay (ELISA) kit (CUSABIO, Wu Han, China).

### 2.6. Western Blotting

After 21 days, the mice were decapitated following anaesthetization with an intraperitoneal injection of 3% sodium pentobarbital (40 mg/kg). The hippocampi of five mice in each group were removed. Proteins were extracted from the hippocampi, and concentrations were detected using a BCA protein assay kit (Beyotime, Shanghai, China). The protein expression of NeuN and GFAP was then measured using Western blotting. The procedure was performed as previously described [8]. In brief, the protein lysates were loaded onto 12% SDS-PAGE for separation, electrotransferred onto PVDF (polyvinylidene fluoride) membranes, and blocked in PBST with 5% nonfat milk. The membranes were incubated with primary antibodies at 4°C overnight. The primary antibodies were GFAP (Abcam, 1 : 2000 dilution), NeuN (Abcam, 1 : 1000 dilution), and anti-*β*-actin (Santa Cruz Biotech, 1 : 2000), respectively. After washing three times for 5 min each in PBST, membranes were incubated with horseradish peroxidase-conjugated secondary antibody. Membranes were developed using an enhanced chemiluminescence detection reagent for 3 min. The optical density of the protein band was measured using the Image J software.

### 2.7. Quantitative Real-Time PCR (qRT-PCR) Detection

The five animals in each group were prepared and anesthetized with 3% sodium pentobarbital, after which the brains were immediately removed and dissected on ice following decapitation. The bilateral hippocampi from each mouse were quickly isolated. Total RNA was extracted using Trizol reagent (Invitrogen). The RNA from each sample was used to synthesize cDNA using High Capacity cDNA Reverse Transcription Kit with Gene Amp PCR System (Applied Biosystems, USA). The sequences for primers were as follows: GAPDH, 5′-GGCAAATTCAACGGCACAGT-3′; 3′-ACGACATACTCAGCACCGGC-5′; GFAP, 5′-CATGCCACGCTTCTCCTTGT-3′; 3′-ATCATCTCTGCACGCTCGCT-5′; NeuN, 5′-GACAACCAGCAACTCCACCC-3′; 3′-GAGCCCCGCTCGTTAAAAAT-5′. qRT-PCR was performed on an ABI ViiA7 Real-Time PCR System (Applied Biosystems, USA) and an SYBR® Green PCR Master Mix in a final volume of 20 *μ*L with the following thermal cycling conditions: 95°C for 1 min, followed by 40 cycles of 95°C for 2 min, 94°C for 10 s, 58°C for 10 s, and 70°C for 38 s. Relative mRNA expression in each sample was calculated as the ratio of mRNA/GAPDH.

### 2.8. Immunofluorescence Staining

The five mice in each group were transcardially perfused with PBS (pH 7.4), followed by 4% paraformaldehyde. Brains were extracted and postfixed overnight in 4% paraformaldehyde at 4°C and then cryoprotected with PBS containing 30% sucrose. Brains were sectioned coronally at 40 um thickness using a Leica MICROTOME. For GFAP and NeuN immunostaining, free-floating brain sections were blocked with 5% normal serum in PBST (0.1%) for 1 h. Sections were then incubated with PBST containing mouse polyclonal anti-GFAP antibody (Millipore, 1 : 200) or mouse polyclonal anti-NeuN (Microtome 1 : 300) for 24 h at 4°C, followed by Alexa Fluor 594-conjugated goat anti-mouse secondary antibody (Invitrogen, 1 : 500) for 2 h at room temperature. For cell-counting experiments, every sixth section between bregma −1.34 mm to −2.46 mm was collected and immunostained for GFAP or NeuN. The sections were mounted and imaged on a confocal laser scanning microscope with 4–20 objectives. The density of GFAP/NeuN^+^ cells in the hippocampal subregions was calculated.

### 2.9. Statistical Analysis

All data are expressed as mean ± standard error of the mean (SEM) and analyzed using the software GraphPad Prism (Version 5.0). The mean values were calculated using one-way analysis of variance (ANOVA) followed by the least significant difference (LSD) test for post hoc comparisons when equal variances were assumed. ANOVA with repeated measures was used to compare the body weight. *P* < 0.05 was considered statistically significant.

## 3. Results

### 3.1. Effects of XYS on CUMS-Induced Changes in Mice Physical State

**Figure 1:**
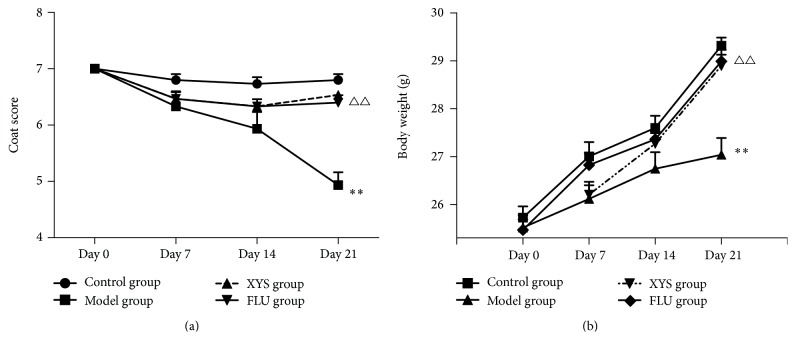
Effects of XYS on CUMS-induced changes in mice physical state. The mice coat state (a) and body weight (b) were measured weekly during the stress period. Data were expressed as mean ± SEM, *n* = 15 per group. ^*∗∗*^*P* < 0.001 versus control; ^△△^*P* < 0.001 versus model.

The CUMS paradigm induced a gradual deterioration of coat state in the model group that did not reach significance after 2 weeks of stress but worsened until the end of the stress-inducing regimen (3 weeks) and was eventually significantly different compared to the model group (*F*(3,56) = 25.08, *P* = 0.000, *P* < 0.001). The physical state of the coat in mice was notably improved after treatment with XYS or FLU compared to the model group (*F*(3,56) = 25.08, both *P* = 0.001, *P* < 0.001, Figure 1(a)).

In order to observe the variation of body weight in the mice subjected to CUMS, body weight in each mouse was measured before the onset of the CUMS regimen and then measured weekly until the end of the CUMS procedure. CUMS mice showed a significant reduction in body weight by the third week (*F*(3,56) = 16.149, *P* = 0.000, *P* < 0.001, Figure 1(b)), while XYS or FLU treatment significantly increased the body weight compared with the model group (*F*(3,56) = 16.149, both *P* = 0.001, *P* < 0.05).

### 3.2. Effects of XYS on CUMS-Induced Depressive-Like Behaviors

**Figure 2:**
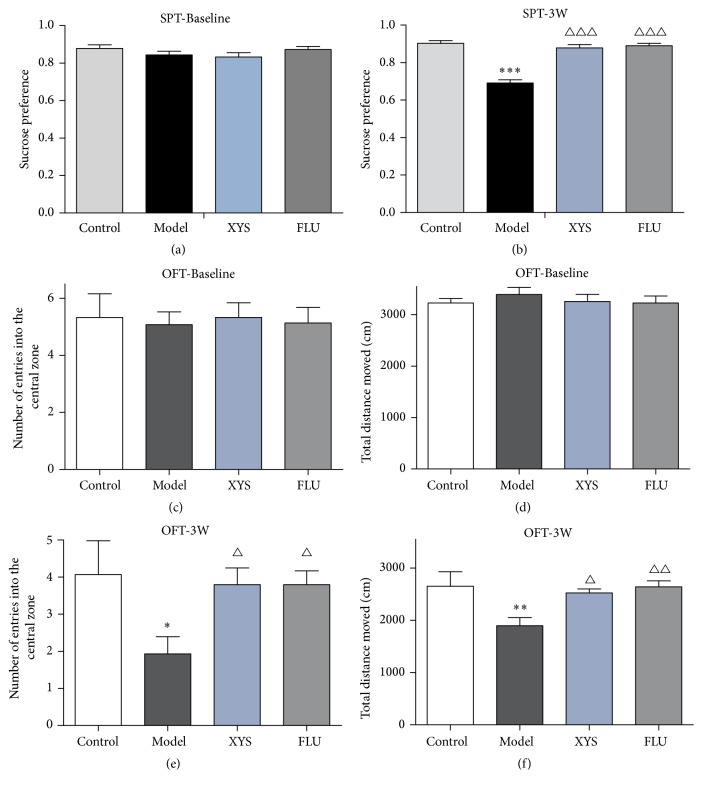
Effects of XYS on CUMS-induced depressive-like behaviors. A battery of behavioral tests was conducted and the following parameters were measured: the number of entries into the center zone (a, c), the total running distance (locomotor activity) (b, d), and sucrose preference (e, f). Data were expressed as mean ± SEM, *n* = 15 per group. ^*∗*^*P* < 0.05, ^*∗∗*^*P* < 0.01, and ^*∗∗∗*^*P* < 0.001 versus control; ^△^*P* < 0.05, ^△△^*P* < 0.01, and ^△△△^*P* < 0.001 versus model.

Anhedonia, which was defined as a reduction in sucrose preference, is the core symptom of depression and is used to evaluate the depressive-like state in rodents [20]. To define the dynamics of the CUMS response, the sucrose preference of each mouse was observed weekly. Initially, mice in all groups had a similar sucrose preference before stress exposure (baseline condition, Figure 2(a)). However, a significant drop in sucrose preference was measured after 3 weeks of stress exposure (*F*(3,56) = 41.379, *P* = 0.000, *P* < 0.001, Figure 2(b)). XYS and FLU treatment effectively reversed these changes and significantly increased the sucrose preference compared with CUMS mice (*F*(3,56) = 41.379, both *P* = 0.000, *P* < 0.001).

In the open field test, all mice exhibited a similar behavioral state before stress exposure (baseline conditions. Figures 2(c) and 2(d)). However, after stress exposure for 21 days, we found that the total distance moved in 5 minutes was significantly different among the groups. The total distance moved by mice in the model group was significantly less (*F*(3,56) = 4.216, *P* = 0.003, *P* < 0.01, Figure 2(f)) than that of mice in the control group. Furthermore, the number of entries into the central zone was also significantly different among groups. In the model group, the number of entries into the central zone was significantly lower (*F*(3,56) = 2.809, *P* = 0.013, *P* < 0.01, Figure 2(e)) than in the control group. FLU treatment attenuated these changes in locomotion patterns and significantly increased the total distance and the number of entries into the central zone (*F*(3,56) = 4.216, *P* = 0.029, *P* < 0.05; *F*(3,56) = 2.809, *P* = 0.004, *P* < 0.01). A similar tendency was also observed in mice treated with XYS (*F*(3,56) = 4.216, *P* = 0.014, *P* < 0.05; *F*(3,56) = 2.809, *P* = 0.029, *P* < 0.05).

### 3.3. Effects of XYS on CUMS-Induced Changes in Neurotrophic Factors

**Figure 3:**
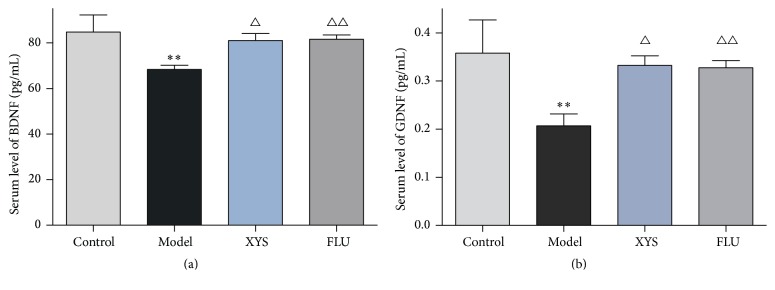
Effects of XYS on CUMS-induced changes in neurotrophic factors. The contents of BDNF (a) and GDNF (b) were measured by ELISA. Data were expressed as mean ± SEM, *n* = 15 per group. ^*∗∗*^*P* < 0.01 versus control; ^△^*P* < 0.05 and ^△△^*P* < 0.01 versus model.

In order to validate the animal model and investigate the animal depressive state induced by CUMS, the contents of brain-derived neurotrophic factor (BDNF) and glial cell line-derived neurotrophic factor (GDNF) in the serum were measured using ELISA. The results in Figure 3 show that CUMS decreased serum BDNF (*F*(3,56) = 2.895, *P* = 0.008, *P* < 0.01) and GDNF levels (*F*(3,56) = 3.068, *P* = 0.008, *P* < 0.01). In contrast, the reductions in BDNF and GDNF could be elevated by XYS (*F*(3,56) = 2.895, *P* = 0.04; *F*(3,56) = 3.068, *P* = 0.025, resp., both *P* < 0.05) and FLU treatment (*F*(3,56) = 2.895, *P* = 0.032, *P* < 0.05; *F*(3,56) = 3.068, *P* = 0.007, *P* < 0.01, resp., Figures 3(a) and 3(b)).

### 3.4. Effects of XYS on CUMS-Induced Changes in Marker Expression in Hippocampal Neurons and Astrocytes

**Figure 4:**
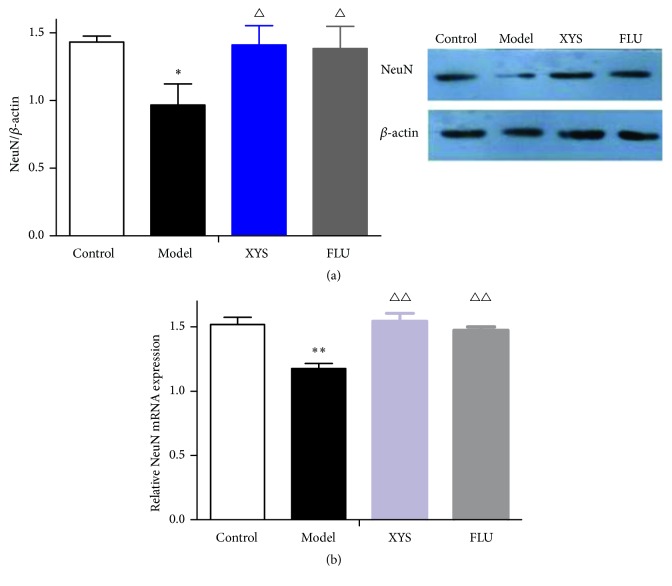
Effects of XYS on CUMS-induced changes in NeuN expressions in the hippocampus. (a) The protein expression of NeuN was measured by Western blotting. (b) The mRNA level of NeuN was measured by qRT-PCR. Data were expressed as mean ± SEM, *n* = 5 per group. ^*∗*^*P* < 0.05 and ^*∗∗*^*P* < 0.01 versus control; ^△^*P* < 0.05 and ^△△^*P* < 0.01 versus model.

**Figure 5:**
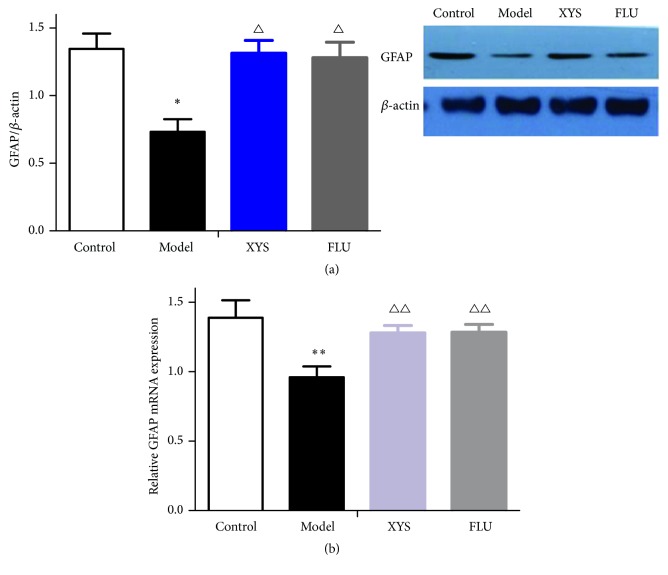
Effects of XYS on CUMS-induced changes in GFAP expressions in the hippocampus. (a) The protein expression of GFAP was measured by Western blotting. (b) The mRNA level of GFAP was measured by qRT-PCR. Data were expressed as mean ± SEM, *n* = 5 per group. ^*∗*^*P* < 0.05 and ^*∗∗*^*P* < 0.01 versus control; ^△^*P* < 0.05 and ^△△^*P* < 0.01 versus model.

**Figure 6:**
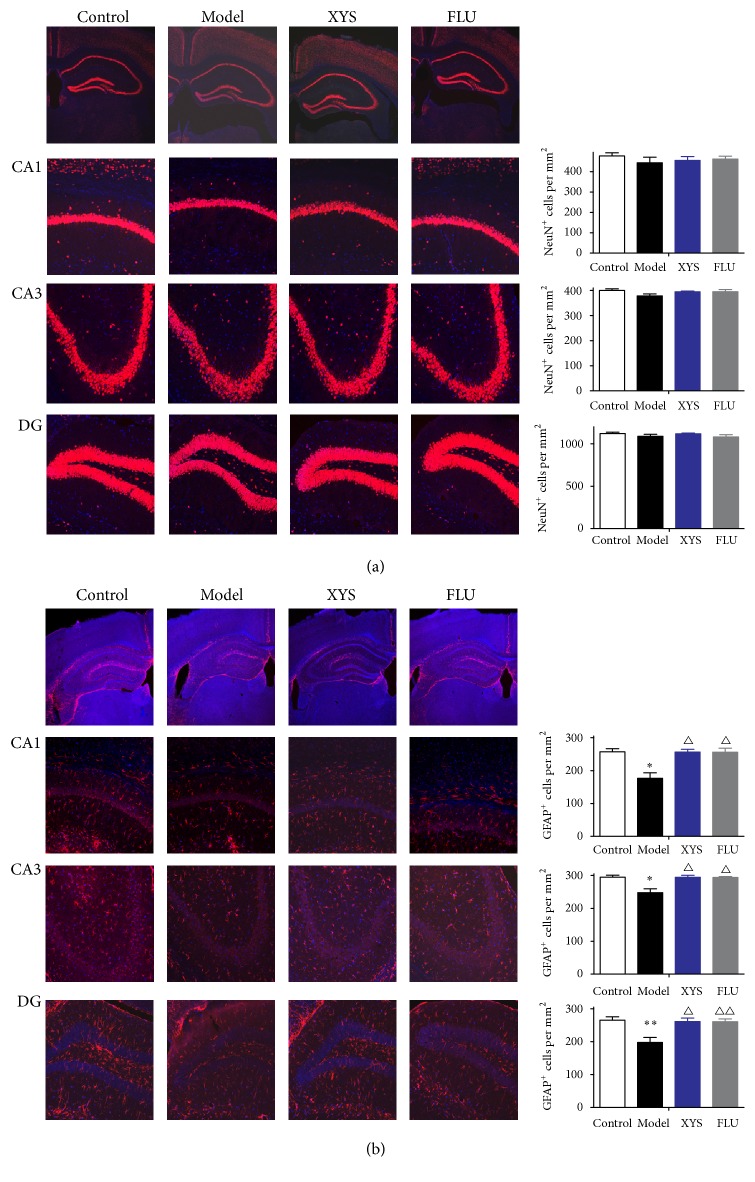
Effects of XYS on CUMS-induced changes in astrocytic cell density in hippocampal subregions. The density of NeuN (a) and GFAP (b) positive cells in the hippocampal subregions CA1, CA3, and DG were determined by immunofluorescence staining. CA1 and CA3: hippocampal subregions of ammon's horn (cornu ammonis); DG: dentate gyrus. Data were expressed as mean ± SEM, *n* = 5 per group. ^*∗*^*P* < 0.05 and ^*∗∗*^*P* < 0.01 versus control; ^△^*P* < 0.05 and ^△△^*P* < 0.01 versus model.

To investigate whether XYS altered marker expression in hippocampal neurons and astrocytes in an animal model of depression, the expression of NeuN and GFAP was measured. First, we analyzed the effects of XYS on the protein and mRNA levels of NeuN and GFAP. As shown in Figures 4 and 5, CUMS exposure resulted in a significant decrease in the protein levels of NeuN (*F*(3,16) = 2.701, *P* = 0.025, *P* < 0.05, Figure 4(a)) and GFAP (*F*(3,16) = 7.825, *P* = 0.015, *P* < 0.05, Figure 5(a)) when compared with the control group. Compared with the model group, XYS treatment significantly elevated these reductions (*F*(3,16) = 2.701, *P* = 0.019; *F*(3,16) = 7.825, *P* = 0.016, both *P* < 0.05) as did FLU treatment (*F*(3,16) = 2.701, *P* = 0.024; *F*(3,16) = 7.825, *P* = 0.012, both *P* < 0.05). As shown in Figures 4(b) and 5(b), a similar tendency was observed in mRNA expression. The mRNA levels of NeuN and GFAP were significantly decreased in CUMS mice when compared with the control group (*F*(3,16) = 12.764, *P* = 0.007; *F*(3,16) = 6.192, *P* = 0.001, both *P* < 0.01). These reductions were also significantly elevated by XYS (*F*(3,16) = 12.764, *P* = 0.009; *F*(3,16) = 6.192, *P* = 0.008, both *P* < 0.01) and FLU administration (*F*(3,16) = 12.764, *P* = 0.002; *F*(3,16) = 6.192, *P* = 0.007, both *P* < 0.01) compared to CUMS mice. We further examined the effects of CUMS and XYS on the expression levels of NeuN and GFAP in the three hippocampal subregions using immunofluorescence staining. As shown in Figure 6(a), the density of NeuN^+^ cells in CA1, CA3, and DG was reduced when compared to the controls, but there were no significant differences. Compared with nonstressed mice, significant reductions in the density of GFAP^+^ cells in three hippocampal subregions were also measured and exhibited significant differences (CA1: *F*(3,16) = 10.727, *P* = 0.016, *P* < 0.05; CA3: *F*(3,16) = 10.076, *P* = 0.039, *P* < 0.05; DG: *F*(3,16) = 8.352, *P* = 0.027, *P* < 0.05, Figure 6(b)). These reductions were effectively reversed by XYS (CA1: *F*(3,16) = 10.727, *P* = 0.017, *P* < 0.05; CA3: *F*(3,16) = 10.076, *P* = 0.041, *P* < 0.05; DG: *F*(3,16) = 8.352, *P* = 0.038, *P* < 0.05, Figure 6(b)) and FLU treatment (CA1: *F*(3,16) = 10.727, *P* = 0.021, *P* < 0.05; CA3: *F*(3,16) = 10.076, *P* = 0.045, *P* < 0.05; DG: *F*(3,16) = 8.352, *P* = 0.035, *P* < 0.05, Figure 6(b)).

## 4. Discussion

The CUMS model was developed based on the stress diathesis model of depression. Most effects of CUMS can be restored by the administration of antidepressant drugs, indicating a strong predictive validity of this depression model [21]. In this model, rats or mice receiving chronic unpredictable stressful stimuli develop core symptoms of major depression, including decreased place preference conditioning, anhedonia, and impaired emotion-like behaviors. Here, we report the effects of CUMS on mouse hippocampus, which is reversed by chronic antidepressant treatment.

The coat state evaluation, unlike the open field test, forced swimming test (FST), or novelty suppressed feeding test (NSF), is a measure that is not associated with depression in humans; however, the coat state assay is the most reliable, prevalent, and well-validated method for the mouse model of depression. In the present study, the CUMS paradigm induced a gradual deterioration of coat state in the model group that did not reach significance by 2-week stress, but worsened until the end of the stress exposure (3 weeks). Similarly, body weight was not significantly different among groups after 7 days of stress exposure. However, after 14 days, CUMS started to affect body weight, leading to a subsequent decrease by day 21. As expected, these alterations in coat state and body weight could be reversed by both XYS and FLU treatment, suggesting that XYS was effective in improving the physical state of mice subjected to CUMS.

To assess the possible depressive behaviors in mice with CUMS, the SPT and the OFT were used. In the SPT, anhedonia is conceptually thought of as “a decreased capacity to experience pleasure of any sort” [22]. In the present study, a significant drop in sucrose preference was measured after 3 weeks of stress exposure and was reversed by both XYS and FLU treatment. The OFT was developed by Denenberg in 1969 [23]. It is an experiment used to assay general locomotor activity levels and to test the emotionality of rodents [24]. Here, the locomotor activity of the model group mice was significantly decreased compared with the control group animals. In addition, the number of entries into the central zone by the model group mice was significantly lower than in the treatment group mice, suggesting that CUMS-induced mice to display depressive-like behaviors. Fortunately, the changes in the open field test could be ameliorated by XYS administration as well as FLU treatment. In agreement with previous studies [8, 14], the present study suggests that CUMS causes a reduction in sucrose consumption and locomotor activity, and XYS is effective for the prevention and treatment of CUMS-induced depression in mice.

To further assess the animal model and depressive state of mice exposed to CUMS, several serum neurotrophic factors were examined. Neurotrophic factors are important signaling molecules in central nervous system (CNS) involved in development and also play roles in the function, survival, and adaptive plasticity of neurons in the brain [25]. BDNF plays a vital role in the regulation of neuronal development as well as in learning and memory [26]. Several studies have demonstrated that BDNF modulates neurogenesis [27–29] which is thought to be involved in the therapeutic efficacy of some antidepressants [30, 31]. One study of postmortem brain samples implicated BDNF as a factor in the pathophysiology of depressive disorders. In another report, BDNF levels were reduced in the hippocampus of depressed patients [32]. Although BDNF is more highly concentrated in brain tissue, it is also present in peripheral tissues, including blood and plasma [33]. It has been reported that BDNF can cross the blood-brain barrier and that brain and serum BDNF levels showed a similar changes during aging in rats, indicating that the latter is a reflection of the former [34]. In the present study, the decreased serum BDNF was measured. Consistent with the clinical report by Karege et al. [34], we found that serum BDNF levels were markedly lower in depressed subjects than in healthy control subjects. The BDNF reduction in the current experiment could be reversed by administration of the antidepressant XYS and FLU. Glial pathology in depressive disorders is well-documented by a number of studies examining the frontolimbic brain regions postmortem. Studies have revealed that glial cell line-derived neurotrophic factor (GDNF) affects cognitive function, including learning and memory [35]. It can exert neuroprotective effects and is responsible for the maintenance and development of central or peripheral neurons [33] and is considered a biomarker for mood disorders [36]. However, it is unclear if peripheral levels of neurotrophins are associated with CNS levels. Diniz et al. found that serum GDNF levels decreased in antidepressant-free patients with MDD [37]. However, plasma GDNF levels were elevated in euthymic patients with MDD and in patients with late-onset depression [38, 39]. This inconsistency in results may be due to confounding effects of gender, age, or concurrent physical illness [40]. Consistent with previous reports, the current study showed that CUMS decreased the serum GDNF levels in depressed mice. Fortunately, chronic treatment with XYS elevated this reduction. In this study, both BDNF and GDNF decreased by 21 d stress, indicating that CUMS successfully induced depression in a mouse model. Additionally, XYS may elevate these reductions to ameliorate the depressive-like behaviors in mice with CUMS.

In the past two decades, NeuN has been observed in most neuronal cell types throughout the nervous system in adult mice and thus serves as a reliable marker of mature neurons [41]. This protein has never been detected in glial cells in vivo, which indicates that it is a specific neuronal marker [42]. In the field of neurological research, NeuN expression levels have been used to directly assess neuronal loss or death, as well as the reappearance of NeuN-positive cells [43, 44] In the current study, the decrease in NeuN mRNA and protein levels in the hippocampi of mice exposed to CUMS was observed; however, these results could not be further confirmed by the immunohistochemical results. These results are further supported by a recent study conducted by Liu et al. who found that the CUMS did not cause significant changes in NeuN-positive cells in the DG, CA1, and CA3 subregions of the hippocampus [45]. This result suggests that the loss of NeuN staining was ascribable to the disappearance of protein immunogenicity rather than a reduction in the NeuN protein expression. A previous study measured the numerical density of neurons in the cerebral cortices of mice exposed to the CUMS procedure which was evaluated after staining. Similarly, no significant differences were also found between the control and CUMS groups [46]. Taken together, neurons expression levels might not be affected in the mice exposed to this CUMS procedure.

Of all of the glial cell types, astrocytes are the most numerous and versatile cells. They play a crucial role in the neuronal microenvironment through regulating such things as synaptic development and neurotransmitter uptake and have been targeted for therapeutic interventions in depression [47]. GFAP, an astrocyte marker, can help astrocytes keep their mechanical strength and shape [48, 49]. Furthermore, GFAP participates in processes linked to cell movement and structure and plays a vital role in astrocyte-neuron communication [50–52]. A previous study showed that decreased GFAP mRNA expression was observed in white and gray matter of the orbitofrontal cortex of subjects experiencing MDD [52]. In subjects with MDD, reductions in the expression of GFAP protein and mRNA, as well as in the density and area fraction of GFAP-immunoreactive (GFAP-IR) astrocytes, could be observed in frontolimbic cortical regions, revealing the dysfunction of astrocytes [52]. In the present study, CUMS decreased mRNA and protein levels, as well the immunoreactivity of GFAP in mouse hippocampus. As expected, XYS treatment could efficiently improve these abnormalities, indicating that astrocyte activity might be enhanced by XYS. The selective serotonin reuptake inhibitor (SSRIs), fluoxetine, is one of the most popular antidepressants used in the clinic [53] and may also involve alterations in NMDA function [54]. Studies evaluating the use of fluoxetine treatment for the prevention of stress-induced decreases in hippocampal GFAP positive astrocytes reported glioprotective effects, which may be relevant to the drug's antidepressant properties [6]. In agreement with previous observations, similar results were also observed in mice treated with FLU that increased GFAP expression in this research. Together, the current study indicates that the traditional Chinese herbal medicine, XYS, showed effects similar to those of fluoxetine for the treatment of depressive disorders.

## 5. Conclusion

**Figure 7:**
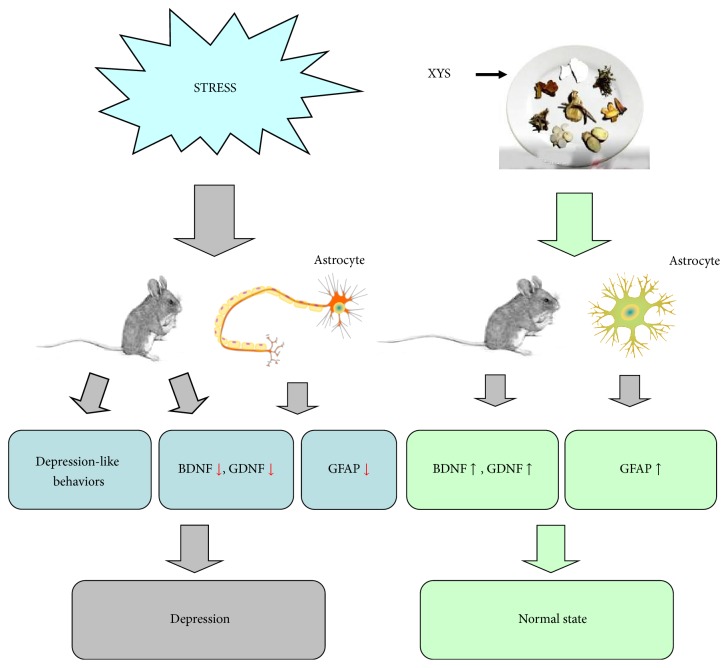
Effects of XYS on CUMS-induced changes in mice hippocampal astrocyte. Chronic unpredictable mild stress (CUMS) led to deficits in depressive-like behaviors in mice, which were improved by XYS administration. CUMS decreased serum BDNF and GDNF levels and reduced the protein and mRNA levels and the immunoreactivity of GFAP in the hippocampus. These abnormalities could be effectively reversed by administration of XYS. These data reveal that chronic administration of XYS elicits an antidepressant-like effect on depressive mice and may normalize the glial fibrillary acidic protein expression in the hippocampus of mice with CUMS.

To our knowledge, no previous studies have directly compared a neuronal specific marker with an astrocytic marker to estimate the effects of the antidepressant XYS on chronically stressed rodents. The current study is the first to demonstrate that changes in GFAP in the hippocampi of mice, caused by chronic stress, can be reversed by XYS. Here, we establish for the first time that the effect of XYS on mouse hippocampus may be of particular relevance to hippocampal astrocytes. Based on the data obtained in this study, a framework for the pathological changes in the hippocampus after exposure to chronic unpredictable mild stress and molecular therapeutic mechanisms is proposed in Figure 7. To further confirm the results of this experiment, future work will include the detection of other neuronal and astrocyte markers in the hippocampi of mice who have experienced 28–48 days of CUMS.

## Figures and Tables

**Table 1:** Procedure of chronic unpredictable mild stress (CUMS).

Day	Restraint stress	Empty cage	Wet and soiled cage	Ice-cold swimming	Food deprivation	Water deprivation	Crowded cage
Monday					8:00 ↓	8:00 ↓	
Tuesday	8:00 ↓ 11:00	8:00 ↓			8:00	8:00	
Wednesday		19:00	8:00 ↓				8:00 ↓
Thursday	8:00 ↓ 11:00		8:00	8:00 ↓ 11:00			8:00
Friday			8:00 ↓		8:00 ↓		
Saturday	8:00 ↓ 11:00		8:00		8:00		8:00 ↓
Sunday		8:00 ↓ 19:00		8:00 ↓ 11:00			8:00
